# Extreme Costs of Mating for Male Two-Spot Ladybird Beetles

**DOI:** 10.1371/journal.pone.0081934

**Published:** 2013-12-05

**Authors:** Jennifer C. Perry, Crystal T. Tse

**Affiliations:** 1 Edward Grey Institute, Department of Zoology, University of Oxford, Oxford, United Kingdom; 2 Jesus College, University of Oxford, Oxford, United Kingdom; 3 Department of Psychology, University of Waterloo, Waterloo, Ontario, Canada; Uppsala University, Sweden

## Abstract

Male costs of mating are now thought to be widespread. The two-spot ladybird beetle (*Adalia bipunctata*) has been the focus of many studies of mating and sexual selection, yet the costs of mating for males are unknown. The mating system of *A. bipunctata* involves a spermatophore nuptial gift ingested by females after copulation. In this study, we investigate the cost to males of mating and of transferring spermatophores in terms of lifespan, ejaculate production and depletion of nutritional reserves. We found that males faced a strong trade-off between mating and survival, with males that were randomly assigned to mate a single time experiencing a 53% reduction in post-mating lifespan compared to non-mating males. This is among the most severe survival costs of a single mating yet reported. However, spermatophore transfer did not impact male survival. Instead, the costs associated with spermatophores appeared as a reduced ability to transfer spermatophores in successive matings. Furthermore, males ingested more food following spermatophore transfer than after matings without spermatophores, suggesting that spermatophore transfer depletes male nutritional reserves. This is to our knowledge the first report of an effect of variation in copulatory behaviour on male foraging behaviour. Overall, our study highlights the advantages of assessing mating costs using multiple currencies, and suggests that male *A. bipunctata* should exhibit mate choice.

## Introduction

Although the costs of mating for females has long been appreciated [1], [2], mating was once thought to be relatively cost-free for males. However, there is now abundant evidence for mating costs for males [3]–[5]. These costs take various forms [5], including decreased ability to acquire future mates and transfer sperm and seminal fluids, decreased survival from increased senescence or extrinsic mortality during mating or mate searching, trade-offs with other physiological functions such as immunity, and the direct metabolic costs of copulation and ejaculate production. The extent of mating costs for males is a significant issue, bearing on mate choice [6], intrasexual competition [7], and sexual conflict [1], and with relevance for theoretical models of sexual selection and honest signaling [8].

The two-spot ladybird beetle *Adalia bipunctata* (Coleoptera: Coccinellidae) has been widely used to investigate numerous questions in mating behaviour and sexual selection, including female preference [9] and mating resistance [10], polyandry [11], nuptial gifts [12], sexually transmitted disease [13], and sperm competition [14]. Yet although the costs of mating for female *A. bipunctata* have been investigated [15], costs for males are entirely unknown. Despite the fact that nuptial gift production can impose high costs in other species [16], the costs to males of producing the spermatophore nuptial gift are likewise unknown. One might expect male mating costs to be considerable in *A. bipunctata*, given that males transfer a relatively large ejaculate (∼4% of male body mass [17]) and mate for several hours [12]. However, male mating costs in other coccinellid beetles are extremely variable, with mating having detrimental, marginal or even positive effects on male lifespan [18], [19].

Here, we investigate male costs of mating and spermatophore transfer in *A. bipunctata* using three approaches that assess costs at distinct levels (following [5]). First, to examine trade-off costs between male reproduction and longevity, we test the effects of mating and spermatophore transfer on male post-mating lifespan by assigning males to a mating or non-mating treatment and recording spermatophore transfer among mating males. Second, we examine trade-offs between current and future reproduction by testing male ability to mate and transfer spermatophores in multiple matings. Finally, to assess the energetic demands placed on males by spermatophore transfer, we examine how spermatophore transfer relates to male feeding rates after mating, and compare the results to a previous finding that mating has no impact on male food intake [20]. We detected costs of both mating and spermatophore transfer for males, including an extreme longevity cost of mating, a decreased ability to transfer sequential spermatophores, and a post-mating increase in male foraging.

## Methods

### Experimental animals

The two-spot ladybird beetle *Adalia bipunctata* (Coleoptera: Coccinellidae) is an aphid predator with Holarctic distribution. Both sexes mate multiply. In the North American population we investigated, mating involves the transfer of a single ejaculate, including seminal fluids that solidify into a spermatophore capsule in the female reproductive tract. Females eject and eat the spermatophore following most matings (60–90%, depending in part on male nutritional state [12], [17]). The ladybirds used in these experiments were of the second and third generation reared in our laboratory, with the original stock obtained from a commercial supplier (Natural Insect Control, Stevensville, Ontario, Canada). Late-instar larvae were separated and the emerging adults grouped by sex. Stock populations were maintained on a mixture of pea aphids (*Acyrthosiphon pisum*, reared on broad bean, *Vicia faba*) and sterilized flour moth eggs (*Ephestia kuehniella* Zeller), a standard diet for this species [21].

### Ethics statement

We did not require a permit to culture *A. bipunctata* because it is native to Canada and not endangered or protected.

### Mating and male survival

To test for an effect of mating and spermatophore transfer on male post-mating lifespan, we randomly assigned virgin males (at least 5 days post-eclosion) to a mating treatment in which they mated once (55 males) or did not mate (19 males). We assigned more males to the mating treatment in order to obtain sufficient males that did or did not transfer a spermatophore during mating. Males were maintained on excess pea aphids prior to the mating trial. To conduct the mating trial, males were introduced individually to a petri dish (50 mm×12 mm), which contained a virgin female for males assigned to the mating treatment. We observed pairs to ensure that copulation occurred (i.e., aedeagus intromission) and lasted at least 30 minutes, the minimum required for sperm transfer [22]. When copulation ended, we moved the male to a new petri dish and observed the female for up to one hour and recorded whether a spermatophore was ejected. Median spermatophore ejection time is 4 minutes post-mating in this population [12]. Non-mating males were similarly moved to a new petri dish. Males were then provided with cotton moistened with 50 µl water daily. Male survival was assessed three times daily from the day following the mating trial until no surviving males remained.

### Sequential mating and spermatophore transfer

We tested the ability of 16 virgin males (at least 5 days post-eclosion) to transfer spermatophores in two sequential matings to virgin females. Males were transferred individually to a petri dish (50 mm×12 mm) containing a virgin female. We observed the pair and when copulation ended, we transferred the male to a new petri dish containing a new virgin female and observed copulation. All copulations exceeded 30 minutes, the minimum required for sperm transfer [22]. We observed females for up to one hour following copulation and noted spermatophore ejection.

### Male post-mating food ingestion

We subjected virgin males to a mating treatment in which they mated once to a virgin female or did not mate, and we then recorded the mass of flour moth eggs (hereafter ‘food’) eaten by males post-mating. This experiment was conducted as part of a larger study on *A. bipunctata* feeding behaviour, and the negligible effect of mating on male food intake has been reported elsewhere [20]. Here we report the effect on male feeding of spermatophore transfer among mated males. The experimental procedure has been described [20]. Briefly, 25 virgin males were introduced to a petri dish (50 mm×12 mm) containing a virgin female. When copulation ended, the male was transferred to a feeding arena (an aluminum weighing boat) containing a known mass of food. After 250 minutes, we transferred the male to a new feeding arena until 24 h post-copulation. We weighed the food remaining in each arena after male removal and generated measures of male feeding over the two intervals by subtraction. We corrected the estimate of the mass of food eaten by subtracting the mean mass loss of food in control feeding arenas that did not contain a ladybird. We monitored mated females for spermatophore ejection for up to one hour post-mating.

### Analyses

We analyzed the effect of mating and spermatophore transfer on male lifespan by a proportional hazards survival model including a single explanatory variable with three levels: not mated, mated with spermatophore transfer, and mated with no spermatophore transfer. We present median lifespan with confidence intervals based on 1000 bootstrap replicates (conducted in Excel 2010, Microsoft, Redmond, Washington, USA). We tested the likelihood of spermatophore transfer in two sequential matings by calculating binomial probabilities. We tested for an effect of spermatophore transfer on male feeding rate by a one-way analysis of variance. Because the sample size was unbalanced, we also analyzed these data with a non-parametric Wilcoxon test. Where appropriate, we confirmed that the data met the assumptions of parametric statistics. Analyses were conducted using JMP v10.0 (SAS Institute, Cary, North Carolina, USA).

### Access to data

Data from this study are available from the corresponding author.

## Results

### Mating and male survival

**Figure 1:**
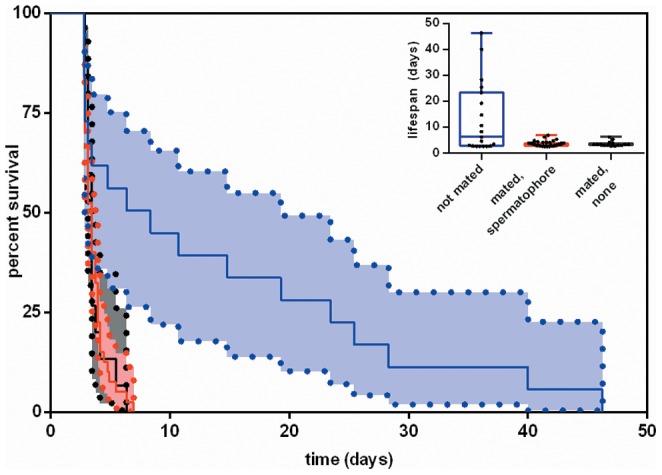
Male post-mating survival and lifespan. Survival curves for males after not mating (blue line) or a single mating in which spermatophore males transferred a spermatophore (red line) or did not (black line). Shaded regions indicate 95% confidence intervals. The inset shows quantile box plots for post mating lifespan in the three groups.

Male survival was strongly influenced by whether males had mated, but not by spermatophore transfer (Figure 1, Table 1). Mated males, regardless of spermatophore transfer, had approximately 4-fold greater risk of death compared to non-mated males (Table 1). One male (in the non-mating treatment) was lost and included as censored data.

**Table 1:** The post-mating lifespan of male ladybirds assigned to a single mating or no mating, in which mated males did or did not transfer a spermatophore.

Treatment	Spermatophore transfer	n	Median days of post-mating lifespan (95% CI)^1^	Hazard ratio (95% CI)^2^	P
not mated		19	7.4 (2.9, 23.5)		
mated	yes	40	3.5 (3.2, 3.8)	4.04 (1.96, 9.25)	0.0002
mated	no	15	3.5 (3.2, 3.5)	3.79 (1.62, 9.40)	

1 Bootstrapped 95% confidence intervals.

2 Results from a proportional hazards survival analysis.

### Sequential mating and spermatophore transfer

All 16 males mated twice within the observation period. Nine of 16 males transferred spermatophores in their first mating. However, only one male transferred a second spermatophore. The binomial probability of observing a value of 1/9 or fewer (given an expected frequency of 9/16) is 0.007, indicating that the likelihood of spermatophore transfer in a second mating is greatly reduced following spermatophore transfer in an initial mating.

Of the 7 males that did not transfer a spermatophore in the initial mating, four transferred a spermatophore in the second mating. This frequency is very similar to that of spermatophore transfer in initial matings and indicates that spermatophore transfer is not more likely in a second mating if it failed in an initial mating.

### Male post-mating food ingestion

The effect of spermatophore ejection on male feeding was similar within both time periods, and we therefore present results for total food ingestion.

**Figure 2:**
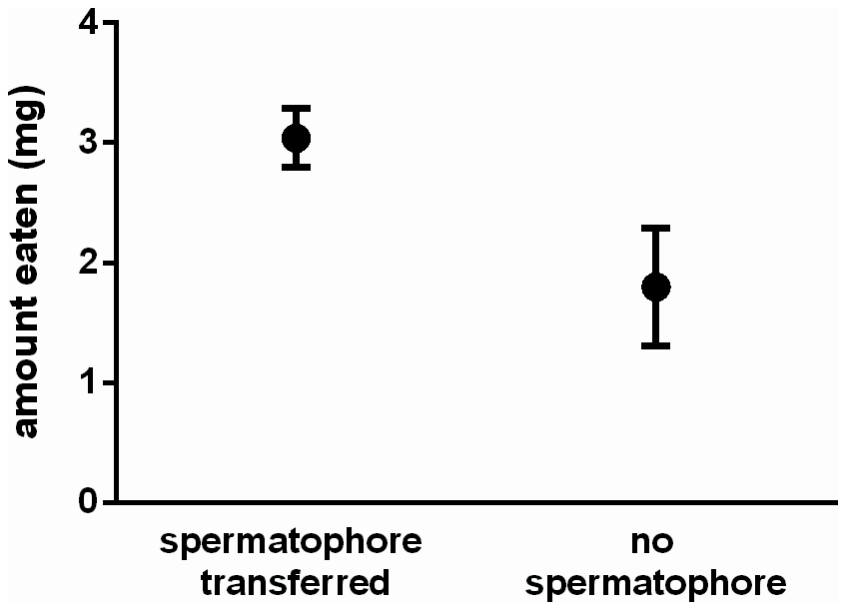
Male post-mating food intake. Food ingestion by males that did or did not transfer a spermatophore in a single mating. Bars indicate standard errors.

We observed spermatophore ejection after 20 of the 25 matings. Males ate approximately 65% more food after transferring a spermatophore than after mating without spermatophore transfer (Figure 2; F_1,22_ = 5.1, P = 0.03). This difference remained significant when analyzed with a non-parametric Wilcoxon test (Z = −2.1, P = 0.03).

## Discussion

Here we have investigated costs associated with mating and spermatophore nuptial gift transfer for male *A. bipunctata*. We find that both are costly, and that costs manifest in distinct ways for each. We demonstrate a dramatic trade-off cost between mating and longevity: median lifespan was reduced 53% by a single mating, among the most severe longevity costs from a single mating that we are aware of from any species. Remarkably, this longevity cost is male-specific: female *A. bipunctata* experience no decline in lifespan from a single or even multiple matings, under similar nutrient-limited conditions [15]. This cost of mating is distinct from the costs associated with spermatophores, as the decrease in lifespan following mating was independent of spermatophore transfer. Instead, spermatophore costs involved a trade-off between current and future reproductive investment, with males rarely able to transfer spermatophores in successive matings. Furthermore, males ingested more food after transferring a spermatophore, indicating that spermatophore transfer depletes male nutritional reserves and alters their post-mating behaviour. This is to our knowledge the first report of the consequences of mating for male foraging behaviour. Below, we discuss each result in the context of the existing literature on the costs of mating and nuptial gifts for males.

### Mating costs

We show that mating entails a survival cost to male *A. bipunctata* that is independent of spermatophore transfer. The magnitude of this cost of mating was extreme. In comparison, males of three other coccinellid beetles experience less severe lifespan effects from mating 5 times, ranging from a ∼5–9% reduction in *Propylea dissecta* to a marginal reduction in *Cheilomenes sexmaculata*, and even a ∼40% increase in *Coccinella septempunctata*, compared to not mating [18], [19]. Further study is required to elucidate the basis of this variation. Although one might suppose that the spermatophore nuptial gift produced by male *A. bipunctata* indicates a more complex and therefore more costly ejaculate, spermatophores are also produced by *C. septempunctata* and sometimes eaten by females [23]. In other insects, experimental studies have reported either no or only minimal reduction in male lifespan from a single mating (in the butterflies *Bicyclus anynana* [24] and *Lethe diana* [25]; in honey locust beetles *Megabruchidius tonkineus* and *M. dorsalis* [26]), or reductions of 13% (in the sepsid fly *Saltella sphondylli* [27]), 21% (in the butterfly *Pieris napi* [24]) and 52% (in the seed bug *Togo hemipterus* [28]). In general, although male longevity costs from mating are widespread [5], the magnitude of cost is extremely variable.

The survival cost of mating cost might be related to the costs of producing sperm and ejaculate components other than the spermatophore capsule, which might be substantial given that these non-spermatophore components represent ∼4% of male body mass in a single mating [17]. Alternatively, the survival costs of mating might stem from the energetic demands of copulation, which typically takes over two hours in this population [12], [17]. Mating costs might also result from energy invested in courtship, as demonstrated in other species [29], but excessive courtship costs are unlikely in *A. bipunctata*. Courtship is very minimal, and it is rare to observe resistance to mating by reproductively mature, non-mated females in good nutritional condition, like those used in these experiments.

Despite the survival costs of mating, we found that males maintained a willingness to mate in two successive matings, even though they were less likely to transfer a spermatophore in a second mating. Some sperm transfer is possible even when spermatophore ejection after mating is not observed (unpublished data), and thus it may benefit males to mate even when unable to transfer a full ejaculate. It is clear that males manage this trade-off between mating opportunities and ejaculate investment in a variety of ways across species, with males of some species delaying re-mating during ejaculate replenishment [30]–[32], and males in other species re-mating and transferring smaller ejaculates [24] or ejaculates depleted in certain components [33]. Our finding that male *A. bipunctata* maintain their willingness to re-mate is in line with findings from other polygynous species in which males are able to mate multiply [34], and also consistent with male behaviour in the ladybird *Coccinella septempunctata*, in which males mate in succession but mating performance (copulation duration and abdominal movements) diminishes over successive matings [35].

### Spermatophore costs

Spermatophore transfer did not impose any detectable longevity costs on males. Instead, costs emerged in two forms. First, our finding of increased male food intake after spermatophore transfer implies that males modify their foraging behaviour to recoup the energetic expenditure associated with spermatophore transfer. Interestingly, the energetic demands of spermatophore transfer do not translate into longevity costs even when males are prevented from recouping that energy through feeding (first experiment; Figure 1). This suggests that investment in spermatophores might instead trade-off with future reproductive investment, which is consistent with our findings, or with other aspects of life history (e.g., immune function) not examined here. We have previously reported a minimal effect of mating itself on male food intake [20], and by comparing Figure 2 of this study with Figure 1(a) of that study, it is evident that food intake by males that did not transfer a spermatophore falls within the range of non-mating males.

A second form of cost was that males had only a limited ability to transfer spermatophores in two consecutive matings, implying that investing in current reproduction comes at the cost of future mating performance. We have discussed above the diverse strategies males use to manage this trade-off. Such declines in performance with additional matings are common [5], but not universal [34].

The costs associated with spermatophore transfer might include those of producing the spermatophore nuptial gift itself, as well as any other ejaculate components correlated with spermatophore transfer. For example, the failure to transfer sufficient quantities of the seminal fluids that form the spermatophore capsule might be associated with lower overall seminal fluid or sperm transfer. However, the failure to transfer a spermatophore does not represent a complete absence of ejaculate transfer, as we have frequently observed sperm transfer in matings that did not involve spermatophore transfer (unpublished data). In general, it is challenging in any species to experimentally isolate the costs of endogenous nuptial gift production (as opposed to gifts that males acquire from the environment, such as prey items [36]) from other costs of mating, because it is difficult to randomly assign nuptial gift production to males. Other correlative evidence for the costs of nuptial gifts comes from orthopterans, including lengthy delays in male re-mating [31], [32], [37], comparative evidence that males take longer to re-mate in species that produce larger nuptial gifts [38], and evidence that spermatophore production trades off with immune function in a cricket [39].

### Conclusions

This study highlights the value of assessing male mating costs using several currencies. We detected a trade-off with longevity for mating itself and a trade off with future reproduction for spermatophore nuptial gift transfer. Furthermore, males increased their rate of foraging after transferring a spermatophore, implying energetic costs. Our findings that both mating and spermatophore transfer are costly for males implies that male *A. bipunctata* should be choosy about their mates. This prediction is at odds with our previous observation that male ladybirds readily attempt mating with female in poor condition [10], [12]. However, even if pre-copulatory male mate choice is indeed limited, male *A. bipunctata* might engage in cryptic mate choice by directing more ejaculate towards high-quality females. Both forms of male mate choice remain open questions in this well-studied mating system.
